# Circulating exosome-derived miR-191-5p is a novel therapeutic biomarker for radiotherapy in esophageal squamous cell carcinoma patients

**DOI:** 10.1007/s10388-025-01116-9

**Published:** 2025-03-10

**Authors:** Huan Wang, Yasunori Matsumoto, Abula Maiyulan, Takeshi Toyozumi, Ryota Otsuka, Nobufumi Sekino, Koichiro Okada, Tadashi Shiraishi, Toshiki Kamata, Hisahiro Matsubara

**Affiliations:** 1 Department of Frontier Surgery, Chiba University Graduate School of Medicine, 1-8-1 Inohana, Chuo-Ku, Chiba-Shi, Chiba Japan; 2 Department of Thoracic Surgery, Liaoning Cancer Hospital & Institute, Shenyang, China

**Keywords:** Esophageal squamous cell carcinoma, Exosome, MiR-191, Radioresistance, Death-associated protein kinase 1, MAPK signaling

## Abstract

**Background:**

Circulating exosomal microRNAs are an easily obtained and minimally invasive biomarker for cancer treatment. Esophageal squamous cell carcinoma (ESCC) is one of the most aggressive carcinomas. It would thus be extremely crucial to predict therapeutic sensitivity and the patient prognosis in advance.

**Methods:**

A search for miRNAs with a therapeutic biomarker in ESCC was performed using the miRNA expression signatures obtained from ESCC plasma exosomes before chemoradiotherapy. miR-191-5p was selected based on a comparison of miRNA signatures and the findings of previous reports. We explored the utility of circulating exosomal miR-191-5p as a prognostic biomarker of chemoradiotherapy along with its target gene, molecular pathway and functions specifically related to radiotherapy in ESCC.

**Results:**

Overexpression of miR-191-5p promoted ESCC cell proliferation, invasion and migration. miRNA-191-5p overexpression promoted cell survival and reduced cell apoptosis after irradiation. Mechanistically, miR-191-5p may downregulate death-associated protein kinase 1 (DAPK1) to induce radiation resistance via the MAPK-JNK pathway. The 5-year progression-free survival rate for ESCC patients who underwent treatment, including radiotherapy with high circulating exosomal miR-191-5p expression was significantly lower than in those with a low expression.

**Conclusion:**

Tumor-derived exosomal miR-191-5p is a potential non-invasive biomarker for predicting the prognosis in esophageal cancer patients after radiotherapy.

**Supplementary Information:**

The online version contains supplementary material available at 10.1007/s10388-025-01116-9.

## Background

Esophageal cancer is the seventh-most common cancer and the sixth leading cause of mortality worldwide [1]. Esophageal squamous cell carcinoma (ESCC) comprises over 90% of all esophageal malignancies in Asia including Japan. Although the treatment of esophageal cancer has progressed in recent years, its prognosis remains poor, with a 5-year survival rate of < 20% [2].

Exosomes are nano-sized extracellular vesicles (50–100 nm in diameter) and detectable in most body fluids, such as plasma, urine, saliva, and ascites [3, 4]. The role of exosomes in cancer progression is gradually being recognized by the study of their various molecular constituents, including proteins and nucleic acids. Tumor-released exosomes have been widely reported to regulate tumor progression, invasion, metastasis, and resistance to chemotherapy [5].

MicroRNAs (miRNAs) are small non-coding RNAs that control gene expression through post-transcriptional regulation [6]. MiRNAs exert various function by targeting different genes. They act as oncogenes or tumor suppressor genes and also play an important role in predicting the therapeutic response. Several studies have suggested that circulating exosomal miRNAs, such as miRNA-21 [7] and miRNA-1246 [8], may be useful as clinical diagnostic or prognostic biomarkers for esophageal cancer. However, the exosomal miRNAs from blood samples associated with therapeutic efficacy have been poorly investigated in ESCC.

Radiotherapy plays a critical role in both neoadjuvant therapy and as radical treatment itself for resectable and unresectable locally advanced esophageal cancer [2]. The identification of molecules involved in ESCC radiosensitivity would be of great significance for the treatment of ESCC. Furthermore, plasma exosome tests that can be performed through blood tests would be minimally invasive by avoiding the need for a biopsy and be extremely versatile.

In the present study, we identified miRNAs related to radiosensitivity in ESCC plasma exosomes. The usefulness of circulating exosomal miR-191-5p as a biomarker for ESCC treatment was examined.

## Material and methods

### Clinical plasma samples

The plasma samples were collected from 67 patients with newly diagnosed ESCC at Chiba University Hospital (Chiba, Japan) between May 2011 and April 2017 and 6 healthy donors. Blood examinations and sampling were performed before treatment. The present study was approved by the Ethics Committee of Graduate School of Medicine, Chiba University (Approval No. 1103). Written informed consent was obtained from all of the patients. The patient characteristics and inclusion criteria were described in Supplementary methods. Progression-free survival (PFS) is defined as the period from the start of treatment until the patient’s cancer progresses or the patient dies from any cause. Disease-specific survival (DSS) was defined as death due to esophageal cancer, starting from the start of treatment, and deaths due to other diseases were treated as censored.

### Global MiRNA expression analyses

miRCURY LNA^™^ microRNA arrays were performed according to the manufacturer’s protocol (Exiqon, Vedbaek, Denmark). MiRNA arrays were performed for the plasma exosomes of ESCC patients before treatment. The tumor regression grade of the primary tumor (TRG-PT) was used to evaluate the therapeutic effect of chemoradiotherapy (CRT). Patients classified as Grade 3 were considered sensitive to CRT (n = 4), while those classified as Grade 0 or 1a were considered resistant to CRT (n = 4). MiRNA arrays were performed in a cohort of eight cases to investigate the difference in the miRNA expression between CRT-sensitive and CRT-resistant patients. The characteristics of eight cases (age, sex, and pathological evaluation findings) are shown in Supplementary Table I.

### Extraction of exosomes from the plasma and transmission electron microscope (TEM) observation

The procedure are described in Supplementary data.

#### MiRNA and mRNA isolation and detection by quantitative real-time polymerase chain reaction (qRT-PCR)

Total RNA was extracted from exosomes using the Total Exosome RNA and Protein Isolation Kit (Invitrogen, Carlsbad, Calif., United States) according to the manufacturer’s protocol. Total cellular RNA was extracted using the miRNeasy Mini Kit (QIAGEN, Hilden, Germany) according to the manufacturer’s protocol. Total RNA was quantified using the Nanodrop Lite (Thermo Fisher Scientific, Santa Clara, CA, USA). The specific stem-looped qRT-PCR primers for miR-191-5p, miR-16, and U6 were designed by Thermo Fisher Scientific. For the detection of the miR-191-5p expression, 10 ng of total RNA was subjected to reverse transcription according to the recommendation of the TaqMan-based microRNA assay. The reaction mixture was incubated at 16 ℃ for 30 min, 42 ℃ for 60 min, and 85 ℃ for 5 min. After cDNA conversion, RT-PCR was performed in triplicate using the MyiQ2 Two-Color Real-Time PCR Detection System (BIO-RAD, Hercules, CA). The reaction mixture was then incubated at 95 ℃ for 10 min, followed by 40 cycles of 95 ℃ for 15 s and 60 ℃ for 1 min. The data were normalized to miR-16 in exosomes and U6 in cells [9].

To measure the expression of death-associated protein kinase 1 (DAPK1), cDNA was generated using a High-Capacity RNA-to-DNA^™^ Kit (Thermo Fisher Scientific, Santa Clara, CA, USA) according to the manufacturer’s protocol. qRT-PCR was performed using SsoFast^™^ EvaGreen Supermix (BIO-RAD, Hercules, CA). The DAPK1-specific primers were as follows: forward, 5’-TGGATATGACAAAGACACATC-3’; reverse, 5’-CTTCATGTCCTTTGACCCAGA-3’. The data were normalized to beta-actin. The fold-changes in the expression of each gene were calculated by the comparative threshold cycle (Ct) method using the formula 2^−ΔΔCt^ [10].

### Cell cultures and miRNA transfection

The human ESCC cell linesTE4, TE6, TE10, TE11, TE15, T-Tn and KYSE-960 were used in the present study. KYSE-960 were obtained from JCRB Cell Bank. TE4, TE6, TE10, TE11, TE15, T-Tn was obtained from Cell Resource Center for Biomedical Research Institute of Development, Aging and Center, Tohoku University, Japan.

The miR-191-5p mimic (CAACGGAAUCCCAAAAGCAGCUG) (Catalog#: AM17000) and negative control mimic were purchased from Thermo Fisher Scientific (Catalog# 4,464,058, Thermo Fisher Scientific, Waltham, MA, USA). The cell lines TE11 and KYSE-960 were seeded into 6-well plates (2.5 × 10^5^ cells per well), and after 24 h, cells were transfected with miR-191-5p mimic or negative control mimic using Lipofectamine^™^ RNAi-MAX (Invitrogen, Carlsbad, Calif., United States) following the manufacturer’s protocol. After 48 h, cells were harvested for further investigation.

### Cell proliferation and colony formation assays

Cells were seeded onto a 96-well plate at a density of 5000 cells per well after transfection with miR-191-5p mimic or negative control mimic in the cell proliferation assay. The cell proliferation was then measured using a Cell Counting Kit-8 (Dojindo Molecular Technologies, Kumamoto, Japan) every 24 h.

Cells were seeded onto a 6-well plate at various cell densities (4 × 10^2^–8 × 10^3^) per well after transfection in the colony formation assay. The plates were then irradiated with a single dose of 0, 2, 4, 6 or 8 Gy X-ray irradiation (MBR-1520R-3; Hitachi) at 24 h after seeding. Following incubation for an additional 10–14 days, the cells were fixed and stained using Diff-Quick Staining (Sysmex Corp., Hyogo, Japan). The number of colonies with > 50 cells was counted. Plating efficiency (PE) was calculated as the number of colonies divided by the number of cells seeded. The survival fraction (SF) was calculated using the following equation:

$${\text{SF}}\, = \,{\text{Colonies Counted}}/{\text{Cells Seeded}}\, \times \,({\text{PE}}/{1}00)$$

The survival curve was derived from a multi-target single-hit model:

$${\text{SF}}\, = \,{1}{-}{1}{-}{\text{exp}}\left( { - {\text{D}}/{\text{D}}_{0} } \right)^{{\text{n}}} ,$$

where D_0_ was defined as the dose that gave an average of one hit per target. The radiation sensitivity enhancement ratio (SER) was measured according to the multi-target single-hit model [11].

### Cell invasion and migration assays

Migration and invasion were detected by the transwell assay. In a 24-well plate, 5 × 10^4^ transfected cells were seeded into the upper chamber (Corning Matrigel Invasion Chamber, BD Biosciences, CA, USA) with FBS-free medium. Medium with 10% FBS was used in the lower chamber. After incubation for 36 h, non-invading cells were removed with a cotton swab from the upper chamber, while the cells on the lower surface were fixed and stained using Diff-Quick Staining (Sysmex Corp, Yokohama, Japan). Pictures of three random fields from triplicate wells were recorded. Migration assays were performed in the same way, except the chambers had no Matrigel coating, and the incubation time was 24 h.

### Cell apoptosis analyses

For the cell apoptosis analyses, cells were irradiated with 0 or 8 Gy X-rays after transfection. After incubation for 24 h, the cells were harvested and washed with PBS, resuspended with 100 μl Annexin V Biding Solution, and incubated with 5 μl of Annexin V FITC and 5 μl of PI solution (Annexin V-FITC Apoptosis Detection Kit; Nacalai Tesque, Kyoto, Japan) at room temperature for 15 min. Finally, 400 μl of Annexin V Biding Solution was added before the analysis. The results were analyzed using the FlowJo software program (TreeStar, Ashland, Oregon, United States).

### Western blot analyses

Western blot procedure and antibodies used in this study are described in Supplementary data.

### Extraction of exosomes from the cell culture medium

The procedure are described in Supplementary data.

### Gene set enrichment analyses

The enrichment analysis of The Cancer Genome Atlas (TCGA) database (https://www.cbioportal.org/datasets) was performed with GSEA v4.0.1. Gene set was obtained from the Molecular Signatures Database v7.2 (http://software.broadinstitute.org/gsea/msigdb). A total of 196 RNAseq data and miRNAseq data points from esophageal cancer patients were used to evaluate the miRNA activities in cancer transcriptomes. Data were divided into miR-191-5p high-expression and low-expression groups based on the median miR-191-5p expression. The C2 and WATANABE_RECTAL_CANCER_RADIOTHERAPY_RESPONSIVE_UP gene sets’ collection was used in this study. False discovery rate (FDR) q values were calculated using 1000 permutations, and a gene set was considered significantly enriched if its normalized enrichment score (NES) has an FDR q below 0.25. A STRING analysis (https://string-db.org/) was used to predict the functional protein association networks of DAPK1.

### Tumor xenografts

Six-week-old female BALB/c Slc-nu/nu mice were used for the present study. Mouse experiments were performed in a specific pathogen-free environment according to the guidelines on animal experiments. The mice were randomly divided into a tumor-bearing group (n = 4) and a tumor-free group (n = 4). For the tumor-bearing group, 10 μl (5 × 106) of TE11 cells suspension were injected directly into back. Seven days after the injection, the tumor volume was measured by calipers, with measurements subsequently repeated every three days. When the average tumor volume reached 300 mm3 (Volume = (width)^2^ × length/2), mice were anaesthetized with isoflurane (4% isoflurane for anesthesia induction, 2% isoflurane for anesthesia maintenance and 2 L/min oxygen flow), and blood samples (about 5 ml to 10 ml)were collected by cardiac puncture. The weight of the mice was 22–26 g at the time of sacrifice. Cervical dislocation was used of euthanizing laboratory mice. Exosomes were then extracted from the plasma for further research.

The present study was performed according to the guidelines on animal experiments and approved by the animal experiment and welfare committee at Chiba University.

### Statistical analyses

The statistical analyses were performed with the SPSS 21 software program (SPSS, Chicago, IL, USA) and the GraphPad Prism 7.04 software program (GraphPad Software, Inc., La Jolla, CA, USA). The difference between two groups was analyzed using unpaired Student’s *t*-test. Comparisons among more than two groups were assessed using a one-way factorial ANOVA. The correlation of miR-191-5p levels in tumor cells and tumor-derived exosomes was examined using Pearson correlation coefficient. Differences in the expression of miR-191-5p in relation to the clinical characteristics were examined using the chi-square test. The Kaplan–Meier method was used to plot survival curves, and the results were compared using the log-rank test. Cox’s proportional hazards regression model was used to analyze the univariate and multivariate survival. P < 0.05 was considered significant.

## Results

### The global miRNA expression analysis for exosomes derived from plasma in esophageal cancer patients

The expression of a subset of 7 miRNAs (miR-628, *p* = 0.0023, miR-363, *p* = 0.0046, miR-191-5p, *p* = 0.025, miR-185, *p* = 0.038, miR-148a, *p* = 0.041, miR-320d, *p* = 0.043, miR-30e, *p* = 0.045) significantly differed between CRT-sensitive and CRT-resistant patients (Table 1). The expression of miR-628, miR-363, miR-185, miR-148a, miR-30e showed significant downregulation in the resistant group, while the expression of miR-191-5p and miR-320d showed upregulation in the resistant group.

**Table 1:** Fold Change and P-values of exosomal miRNA in CRT Resistant group vs Sensitive group

miRname	Fold change	p-value	Regulation
hsa-miR-628-3p	0.68	0.0023	Down
hsa-miR-363-3p	0.35	0.0046	Down
hsa-miR-191-5p	1.39	0.0254	Up
hsa-miR-185-5p	0.53	0.0382	Down
hsa-miR-148a-3p	0.38	0.0405	Down
hsa-miR-320d	2.07	0.0432	Up
hsa-miR-30e-5p	0.60	0.0446	Down

### miR-191-5p expression analysis of ESCC cells and tumor-derived exosomes

**Fig. 1:**
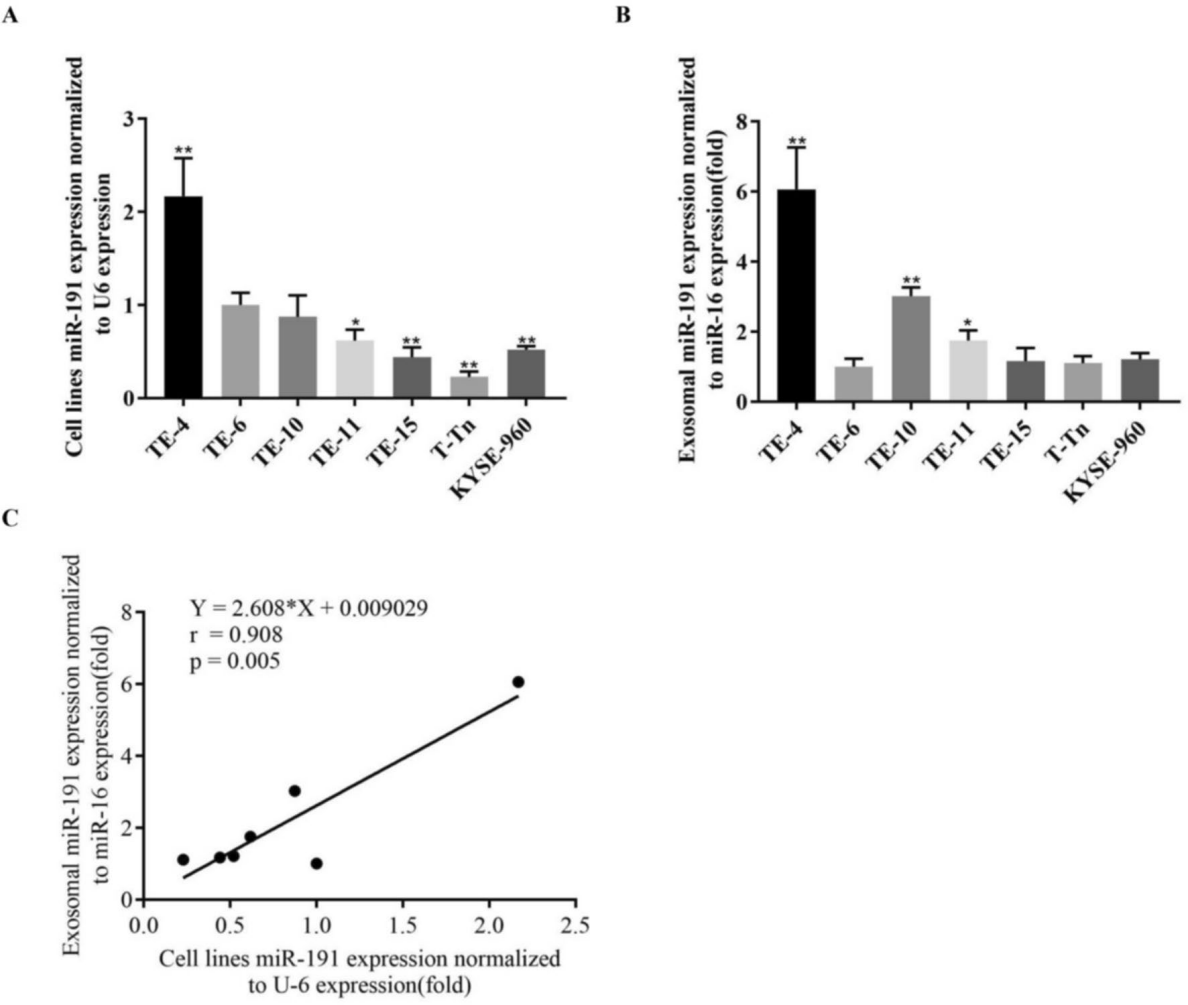
Correlation between the expression miR-191-5p in ESCC cell lines and tumor-derived exosome. A. The expression of miR-191 was detected by PCR in ESCC cell lines and normalized by U6. B. miR-191 expression level was detected by PCR and normalized by miR-16 in exosome-free medium. *P < 0.05 and **P < 0.01 compared with the TE6 group. C. Pearson correlation coefficient (r) and p-value (p) of miR-191-5p expression between ESCC cell lines and tumor-derived exosome

The expression of miR-191-5p in different ESCC cell lines and tumor-derived exosomes were detected by qRT-PCR (Fig. 1 A and B). TE4 showed high expression of miR-191-5p both in the cell line and tumor-derived exosomes. T-Tn showed low expression of miR-191-5p in the cell line and TE6 showed low expression of miR-191-5p in the tumor-derived exosomes, respectively. As shown in Fig. 1C, Pearson correlation demonstrated a strong positive correlation of miR-191-5p levels in tumor cells and tumor-derived exosomes (r = 0.91, p = 0.005). Exosome-enriched protein (CD63, CD81) were confirmed by a Western blot analysis, validating that exosomes were present in the exosome from culture medium (Supplementary Fig. S1).

### *Proliferation and colony formation *in vitro

**Fig. 2:**
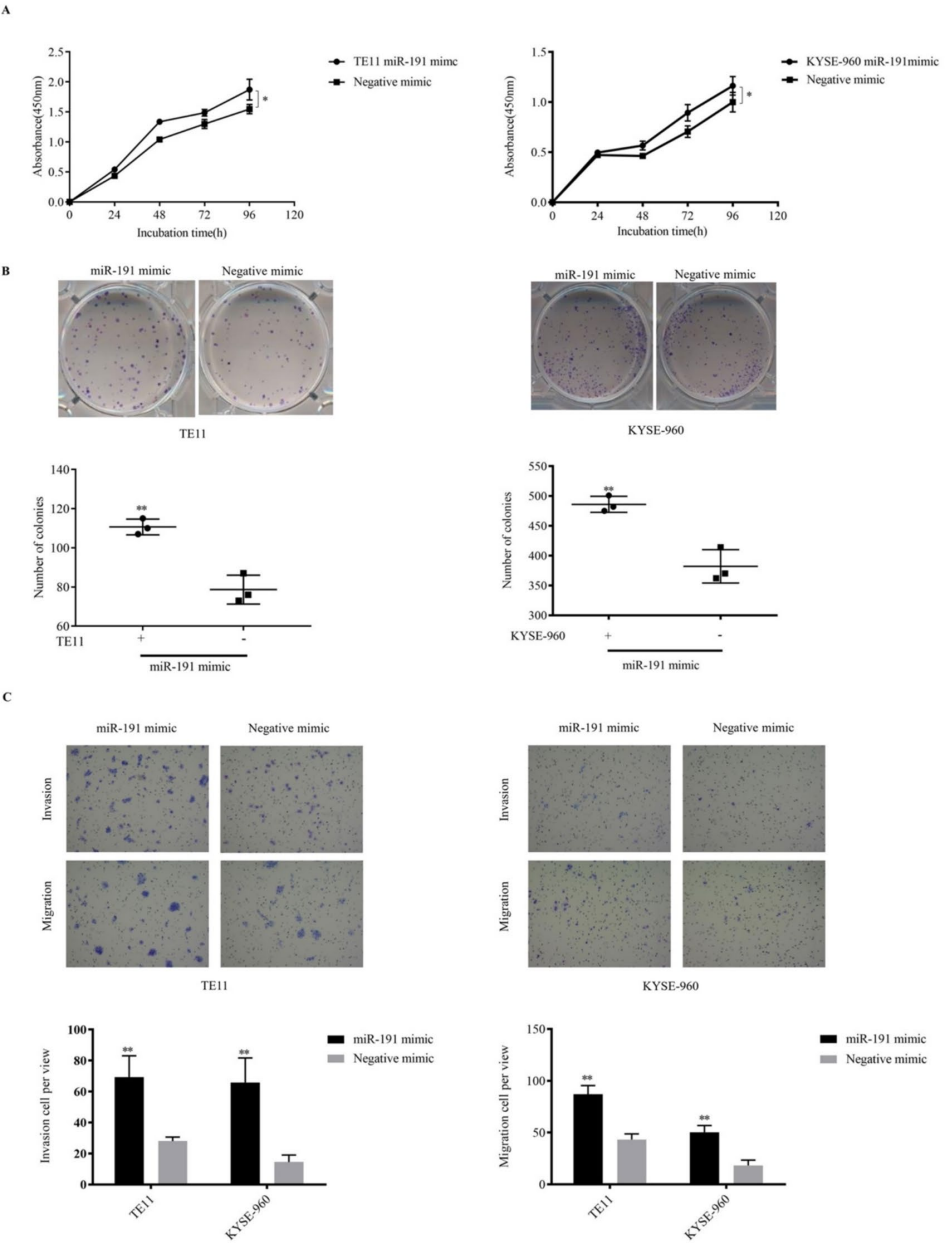
The overexpression of miR-191-5p promoted ESCC proliferation, colony formation, cell invasion and migration. A: The effect of miR-191-5p transfection on ESCC cells proliferation were measured by Cell Counting Kit-8. B: Colony formation assay of TE11 and KYSE-960. The data are presented as the means ± SEM. C: Migration and invasion were detected by transwell assays. The data are presented as the means ± SEM. *P < 0.05 and **P < 0.01 compared with the negative control

The overexpression of miR-191-5p in TE11 and KYSE-960 cell lines after miR-191-5p transfection compared with parent cells was confirmed by qRT-PCR (Supplementary Fig. S2). As shown in Fig. 2A, the cell growth curve was significantly increased after miR-191-5p mimic transfection compared with that in control cells (TE11, p = 0.014; KYSE-960, p = 0.014). In addition, the up-regulation of miR-191-5p dramatically promoted colony formation in ESCC cells (TE11, p = 0.0027; KYSE-960, p = 0.0044, Fig. 2B).

### Cell invasion and migration of ESCC after miR-191-5p transfection

As shown in Fig. 2C, the cell invasion and migration capacity in miR-191-5p overexpression cell lines were significantly higher than in negative controls (Invasion: TE11, mimic vs. control, p = 0.007; KYSE-960, mimic vs. control, p < 0.001. Migration: TE11, mimic vs. control, p < 0.001; KYSE-960, mimic vs. control, p < 0.001).

### Proliferation and apoptosis assays in ESCC cells after irradiation and/or miR-191-5p transfection

**Fig. 3:**
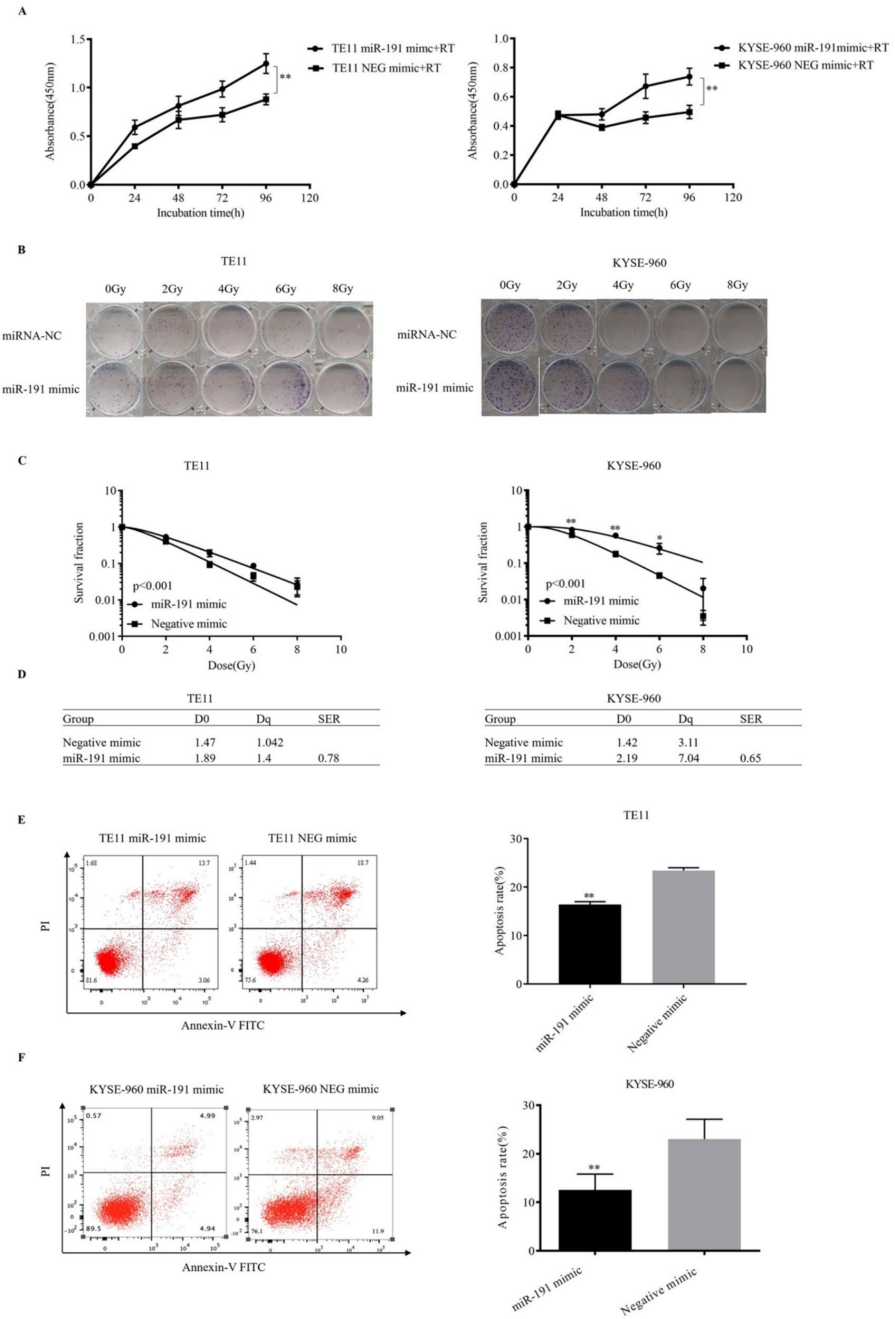
Proliferation, Clonogenic survival assays and apoptosis assays in ESCC cells after irradiation and/or miR-191-5p transfection. A: After 24 h of cell adhesion, cells that had been transfected with mimic and mimic control were treated with 0 or 8 Gy of X-ray irradiation. The proliferation rate was then measured by CCK-8 every 24 h. B: Colony formation assays. The plates were irradiated with a single dose of 0, 2, 4, 6 or 8 Gy X-ray irradiation. C: The surviving fraction was fitted to the multi-target single-hit model. D: D_0_: 37% dose slope; Dq: quasithreshold dose; SER: radiation sensitivity enhancement ratio. E, F: Annexin V/PI double-staining assays were performed to evaluate the cellular apoptosis. *P < 0.05 and **P < 0.01 compared with the negative control

Cells transfected with miR-191-5p showed a higher survival rate than the negative control group after treatment with radiotherapy (RT; TE11, mimic vs. control, p < 0.001; KYSE-960, mimic vs. control, p < 0.001) (Fig. 3A).

The fraction of surviving cells was calculated and compared between miR-191-5p mimic and the negative control. As shown in Fig. 3B and C, cells transfected with miR-191-5p exhibited higher clonogenic survival rates than the negative control under irradiation (0, 2, 4, 6 Gy, p < 0.05). In addition, the miR-191-5p mimics decreased the radiosensitivity of the TE11 (SER = 0.78, p < 0.001) and KYSE-960 cells (SER = 0.65, p < 0.001) (Fig. 3D).

As shown in the Fig. 3E and F, the cellular apoptosis rate of miR-191-5p -transfected TE11 and KYSE-960 cells was significantly lower than in the negative mimic-transfected cells (TE11, mimic vs. control, p = 0.001; KYSE-960, mimic vs. control, p = 0.006).

### Web-based bioinformatics analysis of miR-191-5p target genes and the gene expression analysis of DAPK and MAPK pathway molecules after miR-191-5p transfection

**Fig. 4:**
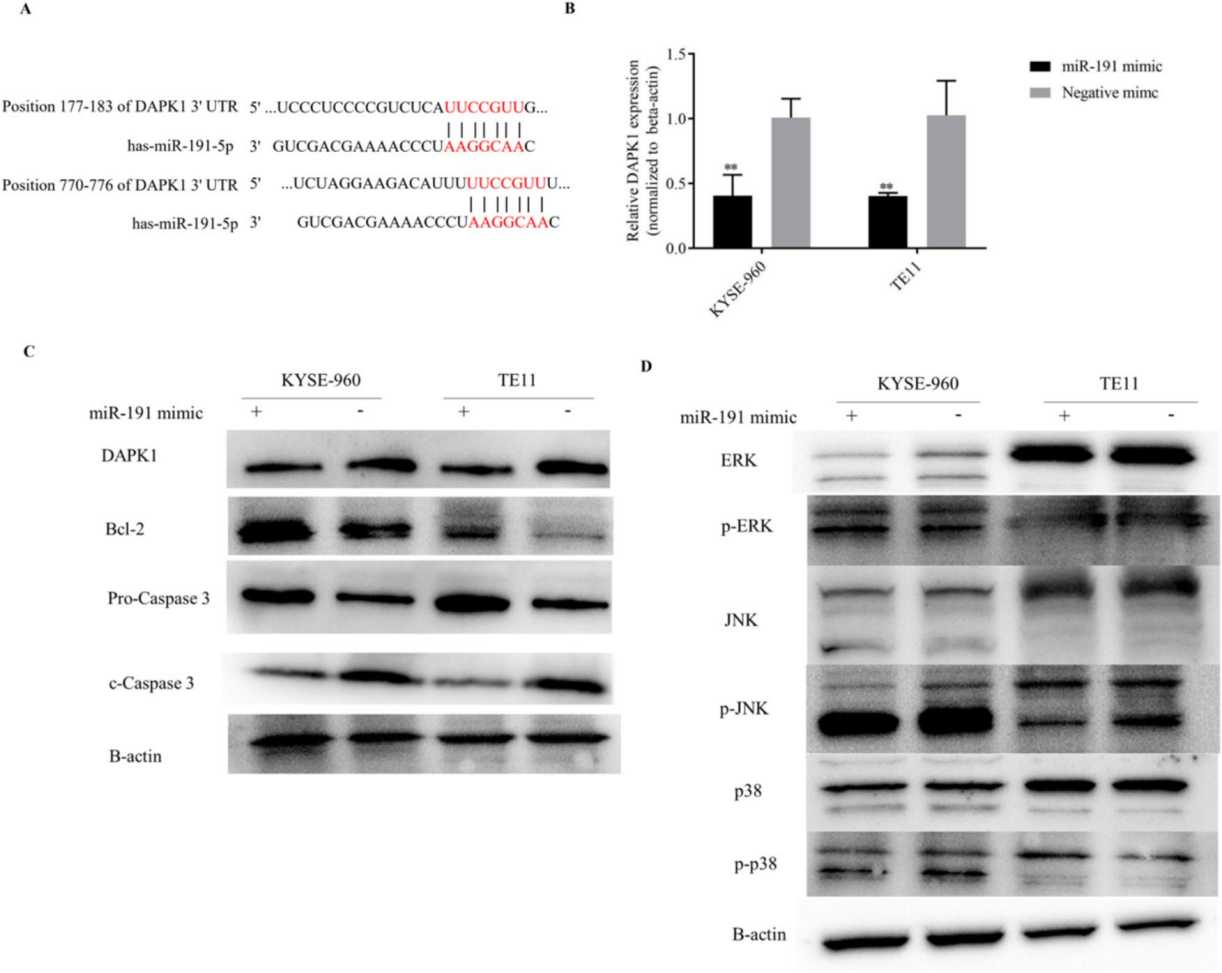
miR-191-5p directly targets DAPK1 to regulate apoptosis through the MAPK pathway. A. Predicted binding sites of miR-191-5p and DAPK1. B. DAPK1 mRNA expression with or without miR-191-5p transfection. C. Western blot analyses of DAPK1 and apoptotic molecules, including Bcl-2 and caspase 3, in TE11 and KYSE-960 cell lines were normalized to beta-actin. D. Western blot analysis of the phospho-active forms of MAPKs (ERK, JNK and p38) in the TE11 and KYSE-960 cell lines were normalized to beta-actin. **P < 0.01 compared with the negative control

According to the bioinformatics analysis, miR-191-5p combined with position 177–183 and 770–776 of the DAPK1 3’ UTR (Fig. 4A). qRT-PCR and a Western blot analysis showed that the DAPK1 mRNA and protein expression was significantly reduced after miR-191-5p transfection (Fig. 4B and C).

**Fig. 5:**
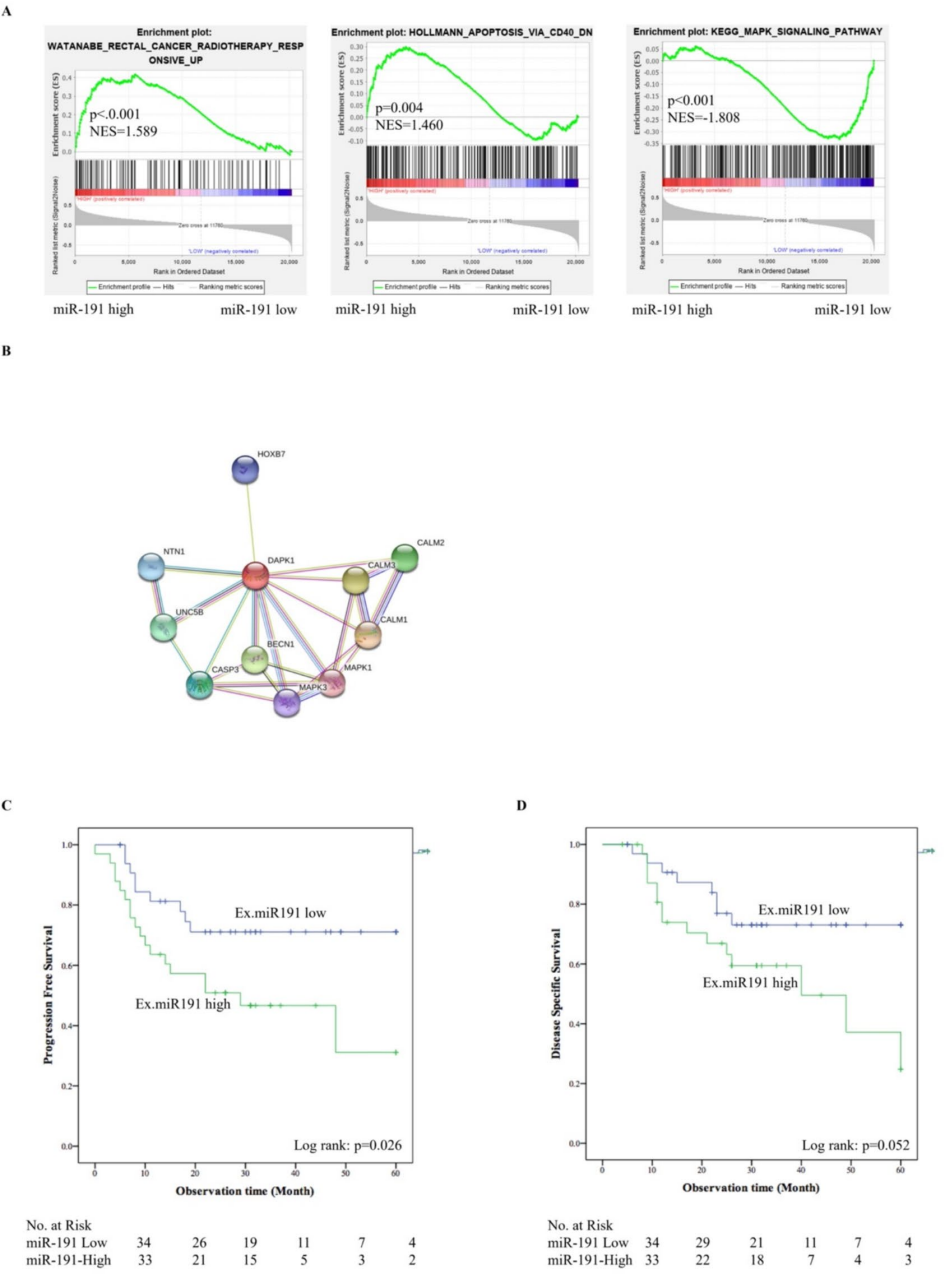
A. The enrichment analysis of The Cancer Genome Atlas (TCGA) database were performed to evaluate the miRNA activities in cancer transcriptomes. B. A STRING analysis was used to predict the functional protein association networks of DAPK1. Kaplan–Meier analysis of the progression-free survival after radiotherapy(C) and the disease-specific survival(D) was performed in 67 esophageal cancer patients according to the miR-191-5p expression. Patients who underwent RT with a low miR-191-5p expression showed a longer PFS than those with a high expression (p = 0.026, log-rank test)

As shown in Fig. 5A, a GSEA analysis of the TCGA database revealed that radiotherapy resistance (p < 0.001; NES = 1.59) and apoptosis resistance (p < 0.001; NES = −1.81) genes were enriched in ESCC patients with a high miR-191-5p expression. In addition, MAPK signaling pathway genes (p = 0.004; NES = 1.46) were enriched in esophageal cancer patients with a low miR-191-5p expression. The result of a STRING analysis indicated that DAPK1 interacted with the MAPK signaling pathway and caspase 3 (Fig. 5B) [12]. The activation status of three MAPK pathways—JNK, extracellular signal-regulated protein kinase (ERK), and p38 mitogen-activated protein kinase (p38 MAPK)—were confirmed with or without miR-191-5p transfection. As the results showed (Fig. 4D), the activation of JNK was inhibited by miR-191-5p. The activation of ERK and p38 were not significantly different between miR-191-5p transfection and the negative control. The overexpression of miR-191-5p inhibited DAPK1 and cleaved-caspase 3 expression. Bcl-2 was up-regulated by miR-191-5p (Fig. 4C).

### *miR-191-5p expression analysis of tumor-derive exosome *in vivo

Exosomes were extracted from plasma of tumor-bearing and tumor-free mice. The expression of exosomal miR-191-5p was significantly higher in the tumor-bearing group than in the tumor-free group (p = 0.0014) (Supplementary Fig. S3A).

### Exosome extraction and the exosomal miR-191-5p expression analysis of ESCC patients’ plasma

Exosomes were confirmed by TEM (Supplementary Fig. S3B). As shown in Fig. S3C, exosomal miR-191-5p was significantly overexpressed in esophageal cancer patients compared with healthy donors according to qRT-PCR (p = 0.048).

### Progression-free survival (PFS) and Disease-specific survival (DSS) of the ESCC patients after RT

The clinical characteristics of the patients are shown in Supplementary Table II. Patients were divided into a high-expression group (n = 33) and low-expression group (n = 34) according to the median expression of miR-191-5p (cut-off value ≥ 2.5). The clinical characteristics did not marked differ between the two groups. Patients who underwent RT with a low miR-191-5p expression showed a longer PFS than those with a high expression (p = 0.026, log-rank test) (Fig. 5C). The disease-specific survival rate of the low-expression group was higher than in the high-expression group, although not to a significant degree (p = 0.052, log-rank test) (Fig. 5D). The univariate and multivariate analyses of the factors associated with the PFS of RT patients indicated that the exosomal miR-191-5p expression and cStage were independent prognostic factors (Table 2).

**Table 2:** Hazard ratio of clinical factors for the PFS of esophageal cancer (Cox’s regression hazard model)

Factor	p-value(univariate)	p-value(multivariate)	Hazard radio(95%CI)
Age ≥ 68	0.845		
male vs female	0.397		
Pathological type (poor vs others, SCC)	0.891		
Location (Thoracic vs others)	0.386		
Stage (Stage III/IV vs Stage I/II)	0.043	0.033	8.974 (1.191–67.593)
Exosomal miR-191-5p (High vs Low)	0.022	0.01	2.929 (1.296–6.922)

### Clinical and pathological effect of CRT between high and low miR-191-5p expression groups

In a cohort of 59 patients who underwent chemoradiotherapy (CRT) followed by esophagectomy (excluding those who received CRT alone or salvage endoscopic submucosal dissection), CRT failure (Grade 1a) occurred in 25.9% of patients with high levels of miR-191 and in 13.8% of those with low levels of miR-191, as detailed in Supplementary Table III and Supplementary Fig. S4. However, this difference was not statistically significant (P = 0.3224, Fisher’s exact test).

## Discussion

Concurrent chemoradiotherapy has been the preferred approach for patients with esophageal squamous cell carcinoma, aiming to achieve downstaging prior to surgery [13]. Based on findings from the CROSS trial, the 10-year overall survival benefit was 13% (38% versus 25%). Neoadjuvant chemoradiotherapy significantly reduced the risk of death from esophageal cancer, with a hazard ratio of 0.60 (95% CI 0.46–0.80) [14]. It would thus be extremely crucial to predict therapeutic sensitivity and the ESCC patient prognosis in advance. The present study therefore explored the molecular factors of the exosome responsible for conferring radioresistance in order to develop new strategies to increase patients’ sensitivity to irradiation.

In this study, we found 7 exosome-derived miRNAs had differently expressed between CRT-sensitive and CRT-resistant patient. We detected the expression of each exosomal miRNA in few patient plasma samples as a pilot study. From the result, miR-191-5p was stable expression than the other miRNAs. Thus, we decide to focuse on the study of miR-191-5p. Accumulating data in recent years has indicated that miR-191-5p is abnormally expressed in more than 20 different cancers and is a major player in some of these entities [20].

In the present study, the overexpression of miR-191-5p promoted the cell survival and decreased cell apoptosis after irradiation. A clonogenic survival assay indicated that miR-191-5p decreased radiosensitivity 0.78- and 0.65-fold in TE11 and KYSE-960 cells, respectively. Therefore, these results indicate that miR-191-5p synergistically elicits radiation resistance by inhibiting radiation-induced apoptosis.

Liquid biopsy, a minimally invasive method for sampling and analyzing tumor-derived substances, shows great promise in predicting therapeutic responses and serving as diagnostic biomarkers. Circulating blood, due to its rich molecular content and accessibility, has emerged as the primary sampling source for liquid biopsy, attracting substantial attention in recent years [15]. Emerging evidence suggests that exosomes, particularly those secreted by tumor cells carrying oncogenes, mediate intercellular communication and contribute to cancer progression, metastasis, and radioresistance [16, 17]. Circular RNAs (circRNAs), a novel class of non-coding RNAs, play significant roles in biological processes related to tumorigenesis [18]. Recent studies indicate that circular RNAs (circRNAs) within exosomes may play a pivotal role in the transfer of biological activity and gene regulation by interacting with microRNAs (miRNAs) in their originating cells. Specifically, circRNAs may function as miRNA sponges in recipient cells[19]. However, the mechanisms by which circRNAs in exosomes modulate miR-191-5p remain poorly understood and still needs to be further investigation.

DAPK1 acts as a positive mediator of apoptosis induced by many apoptotic signals, including DNA-damaging agents, different death stimuli [21]. Previous studies have shown that DAPK1 is a tumor-suppressive gene and is suppressed in various cancers [22]. GSEA results showed that the MAPK pathway was upregulated in the miR-191-5p low-expression group and suppressed in the miR-191-5p high-expression group. DAPK1 has also been found to be a key factor regulating the MAPK pathway [21]. In a previous study, miR-191-5p inhibited TNF-α-induced apoptosis of ovarian endometriosis and endometrioid carcinoma by targeting DAPK1 [23]. DAPK1 acts as an upstream activator of JNK, which is the vital downstream signal of the MAPK pathway [24]. The inhibition of JNK activation, not ERK or p38, by miR-191-5p transfection has been confirmed in our study. Taken together, these previous findings provide important insight into the fact that miR-191-5p directly targets DAPK1 to regulate irradiation-induced apoptosis by decreasing the JNK activation. In the present study, we reported that DAPK1 act as a direct target of miR-191-5p and described its potential signaling pathway in ESCC.

The difference in the expression of miR-191-5p between tumor cells and tumor cell-derived exosomes was investigated in vitro in the present study. Interestingly, a strong positive correlation was seen for the expression of miR-191-5p in tumor cells and tumor-derived exosomes. The miR-191-5p expression in tumor-derived exosome was increased with the increase of its expression in tumor cell. These results suggest that miR-191-5p may be secreted into the circulation in great amounts via exosomes. Some studies have reported that in the serum, miR-191 was the prognostic marker of such as lung cancer [25], gastric cancer [26]. Furthermore, the plasma exosomal miR-191-5p expression may be a better reflection of a cancer-bearing patient’s status than the miR-191-5p expression in tumor tissue assessed by a biopsy. However, further investigations on these points are still needed.

Patients who underwent RT with a low expression of miR-191-5p in circulating exosomes showed a longer PFS than those with a high expression of miR-191-5p. While there is no notable evidence that miR-191-5p affects the disease-specific survival of esophageal cancer, miR-191-5p was reported to be frequently overexpressed in ESCC tissues and significantly related to an advanced clinical stage, metastasis, and poor survival rate of ESCC [27]. Therefore, circulating exosomal miRNA-191 may act as a potential prognostic biomarker in ESCC patients after RT. In patients who underwent chemoradiotherapy (CRT) followed by esophagectomy (excluding those who received CRT alone or salvage endoscopic submucosal dissection), those with high levels of exosomal miR-191-5p appeared to exhibit greater resistance to CRT compared to those with low levels of exosomal miR-191-5p, although this difference did not reach statistical significance (25.9% vs 13.8%, P = 0.3224). These findings suggest that exosomal miR-191-5p levels may potentially influence treatment efficacy in clinical practice, although further investigation is warranted.

However, this study has potential limitations. Our study has limitations inherent to retrospective studies. The patient population was small and only associated with a single institution which makes it subject to information biases. In this study, we focus on only the gain of function of miR-191-5p rather than the loss of function. Maybe the siRNA knocked down experiment to negatively prove the role of miR-191-5p in the cell proliferation is better. The role of miR-191-5p in the inter-cell communication mechanisms is still need more experiment. Additionally, we found miR-191-5p regulates irradiation induced apoptosis by decreasing the JNK activation. However, after irradiation and miR-191-5p transfection, the changes of activations of JNK, ERK, and p38 are not evaluated. We did not use luciferase reporter gene system to identify whether DAPK1 is a downstream molecular of miR-191-5p in this study due to the budget issue. However, in the previous study, DAPK1 was confirmed by a luciferase reporter worked as a downstream molecular of miR-191-5p in ovarian endometriosis and endometrioid carcinoma [23]. Whether miR-191-5p could serve as a potential prognostic biomarker for ESCC patients after RT needs further multi-central clinical studies. Additionally, further studies will be required to obtain more evidence concerning the relationship between DAPK1 and the JNK signaling pathway.

## Conclusion

In conclusion, the tumor-derived exosome miR-191-5p expression was suggested to be a useful non-invasive biomarker for predicting the prognosis in patients with esophageal cancer after radiation therapy. In addition, the present study provides evidence that miR-191-5p downregulates DAPK1 expression to regulate irradiation-induced apoptosis by decreasing the JNK activation.

## Supplementary Information

Below is the link to the electronic supplementary material.

Supplementary file1 (DOCX 18 KB)

Supplementary file2 (DOCX 17 KB)

Supplementary file3 (DOCX 16 KB)

Supplementary file4 (DOCX 730 KB)

Supplementary file5 (DOCX 21 KB)

## Data Availability

All data generated or analyzed during this study are included in this published article and its additional files. Original data generated of miRNA microarray was published in GEO (GSE195974, https://www.ncbi.nlm.nih.gov/geo/query/acc.cgi?acc=GSE195974). Further details are available on request.
