# Supplementary material for: Circulating exosome-derived miR-191-5p is a novel therapeutic biomarker for radiotherapy in esophageal squamous cell carcinoma patients
Source: Esophagus. 2025 Mar 10;22(3):454–66. doi: 10.1007/s10388-025-01116-9 (PMC12167317; doi:10.1007/s10388-025-01116-9)
Supplement: Supplementary file 1 — Supplementary file1 (DOCX 18 KB) [file 10388_2025_1116_MOESM1_ESM.docx]

Supplementary Table I. Demographics and clinicopathological characteristics in esophageal squamous cell carcinoma patients (UICC 8th)

|  | CRT-sensitive patients | | | |  | CRT-resistant patients | | | |
| --- | --- | --- | --- | --- | --- | --- | --- | --- | --- |
|  | 1 | 2 | 3 | 4 |  | 5 | 6 | 7 | 8 |
| Age | 69 | 57 | 66 | 68 |  | 73 | 63 | 70 | 73 |
| Sex | M | M | M | M |  | F | M | M | M |
| Location of the tumor | Ut | Mt | Mt | Mt |  | Lt | Mt | Mt | Lt |
| G category | N.A | 2 | N.A | N.A |  | 1 | 2 | 3 | 1 |
| cT category | 4b | 3 | 1b | 4b |  | 3 | 3 | 1b | 3 |
| cN category | 2 | 3 | 2 | 3 |  | 2 | 2 | 4 | 2 |
| cStage | 4a | 4a | 2 | 4a |  | 3 | 3 | 4a | 3 |
| pT category | X | X | X | X |  | 3 | 3 | 1b | 3 |
| pN category | 0 | 0 | 2 | 0 |  | 3 | 0 | 0 | 0 |
| pStage category | X | X | X | X |  | 3 | 2 | 1 | 2 |
| Pathological Grade | 3 | 3 | 3 | 3 |  | 1a | 1a | 1a | 1a |
