# Supplementary material for: Circulating exosome-derived miR-191-5p is a novel therapeutic biomarker for radiotherapy in esophageal squamous cell carcinoma patients
Source: Esophagus. 2025 Mar 10;22(3):454–66. doi: 10.1007/s10388-025-01116-9 (PMC12167317; doi:10.1007/s10388-025-01116-9)
Supplement: Supplementary file 2 — Supplementary file2 (DOCX 17 KB) [file 10388_2025_1116_MOESM2_ESM.docx]

| Supplementary Table II. Demographics and clinicopathological characteristics in esophageal squamous cell carcinoma patients (UICC 8th) | | | |
| --- | --- | --- | --- |
| Exosomal miR-191-5p | high (N=33) | low (N=34) | p |
| Age | 66.2±8.59 | 66.2±8.59 | 0.452 |
| Sex,n |  |  |  |
| Male/ Female | 27/ 6 | 32/2 | 0.15 |
| Location of the tumor |  |  |  |
| Ce/ Ut/ Mt/ Lt/ Ae/ X | 3/ 9/ 8/ 9/ 1/ 3 | 2/ 6/ 15/ 10/1/ 0 | 0.309 |
| G category, n |  |  |  |
| G1/G2/G3/N.A | 6/ 7/ 2/ 19 | 2/ 10/ 6/ 15 | 0.173 |
| cT category, n |  |  |  |
| cT1b/ 2/ 3/ 4a/ 4b/ X | 6/ 1/ 17/ 1/ 7/ 1 | 5/ 1/ 10/ 2/ 16/ 0 | 0.24 |
| cN category, n |  |  |  |
| cN0/ 1/ 2/ 3 | 5/ 8/ 11/ 8 | 4/ 6/ 12/ 12 | 0.758 |
| cStage, n (%) |  |  |  |
| cStage 0/ 1/ 2/ 3/ 4a/ X | 4/ 3/ 15/ 10/ 1 | 3/ 2/ 9/ 20/ 0 | 0.187 |
| Treatment, n |  |  |  |
| Surgery after RT/CRT | 30 | 32 | 0.673 |
| RT/CRT | 3 | 2 |  |
| pT category, n |  |  |  |
| pT0/ 1a/ 1b/ 2/ 3/ X | 10/ 2/ 5/ 3/ 12/ 2 | 10/ 2/ 5/ 3/ 12/ 2 | 0.539 |
| pN category, n |  |  |  |
| pN0/ 1/ 2/ 3/ X | 18/ 6/ 4/ 2/ 4 | 11/ 6/ 9/ 3/ 4 | 0.434 |
| pStage, n (%) |  |  |  |
| pStage 1/ 2/ 3/ 4a/ X | 10/ 3/ 9/ 7/ 1/ 4 | 7/ 1/ 7/ 6/ 0/ 12 | 0.233 |
