# Supplementary material for: Circulating exosome-derived miR-191-5p is a novel therapeutic biomarker for radiotherapy in esophageal squamous cell carcinoma patients
Source: Esophagus. 2025 Mar 10;22(3):454–66. doi: 10.1007/s10388-025-01116-9 (PMC12167317; doi:10.1007/s10388-025-01116-9)
Supplement: Supplementary file 3 — Supplementary file3 (DOCX 16 KB) [file 10388_2025_1116_MOESM3_ESM.docx]

| Supplementary Table III.  exosomal miR-191-5p and histological Grade by CRT followed by esophagectomy | | |
| --- | --- | --- |
| TRG-PT | miR-191-5p High（n=29） | miR-191-5p Low(n=30) |
| Grade 1a | 7(24.1%) | 4(13.3%) |
| Grade 1b | 2(6.9%) | 4(13.3%) |
| Grade 2 | 9(31.0%) | 12(40.0%) |
| Grade 3 | 9(31.0%) | 9(30.0%) |
| unknown | 2(6.9%) | 1(3.3%) |
|  |  |  |
| TRG-PT | miR-191-5p High（n=27） | miR-191-5p Low(n=29) |
| Grade 1a | 7 | 4 |
| ≥Grade 1b | 20 | 25 |
