# Supplementary material for: Circulating exosome-derived miR-191-5p is a novel therapeutic biomarker for radiotherapy in esophageal squamous cell carcinoma patients
Source: Esophagus. 2025 Mar 10;22(3):454–66. doi: 10.1007/s10388-025-01116-9 (PMC12167317; doi:10.1007/s10388-025-01116-9)
Supplement: Supplementary file 4 — Supplementary file4 (DOCX 730 KB) [file 10388_2025_1116_MOESM4_ESM.docx]

Supplementary material for

Circulating exosome-derived miR-191-5p is a novel therapeutic biomarker for radiotherapy in esophageal squamous cell carcinoma patients.

Huan Wang^12^, Yasunori Matsumoto^1^, Abula Maiyulan^1^, Takeshi Toyozumi^1^, Ryota Otsuka^1^, Nobufumi Sekino^1^, Koichiro Okada^1^, Tadashi Shiraishi^1^, Toshiki Kamata1 and Hisahiro Matsubara^1^

1. Department of Frontier Surgery, Chiba University, Chiba, Japan.

2. Department of Thoracic Surgery, Cancer Hospital of China Medical University, Liaoning Cancer Hospital & Institute, Shenyang, China.

**Supplementary figure**

**
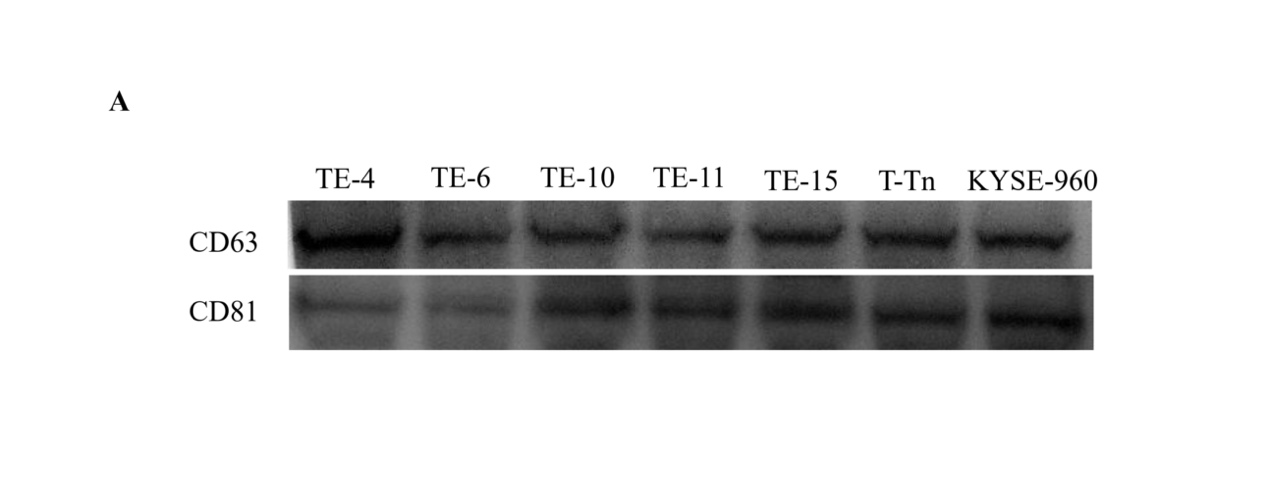
**

**Figure S1.** A. Exosome markers(CD63, CD81) were used to identify exosomes.

**
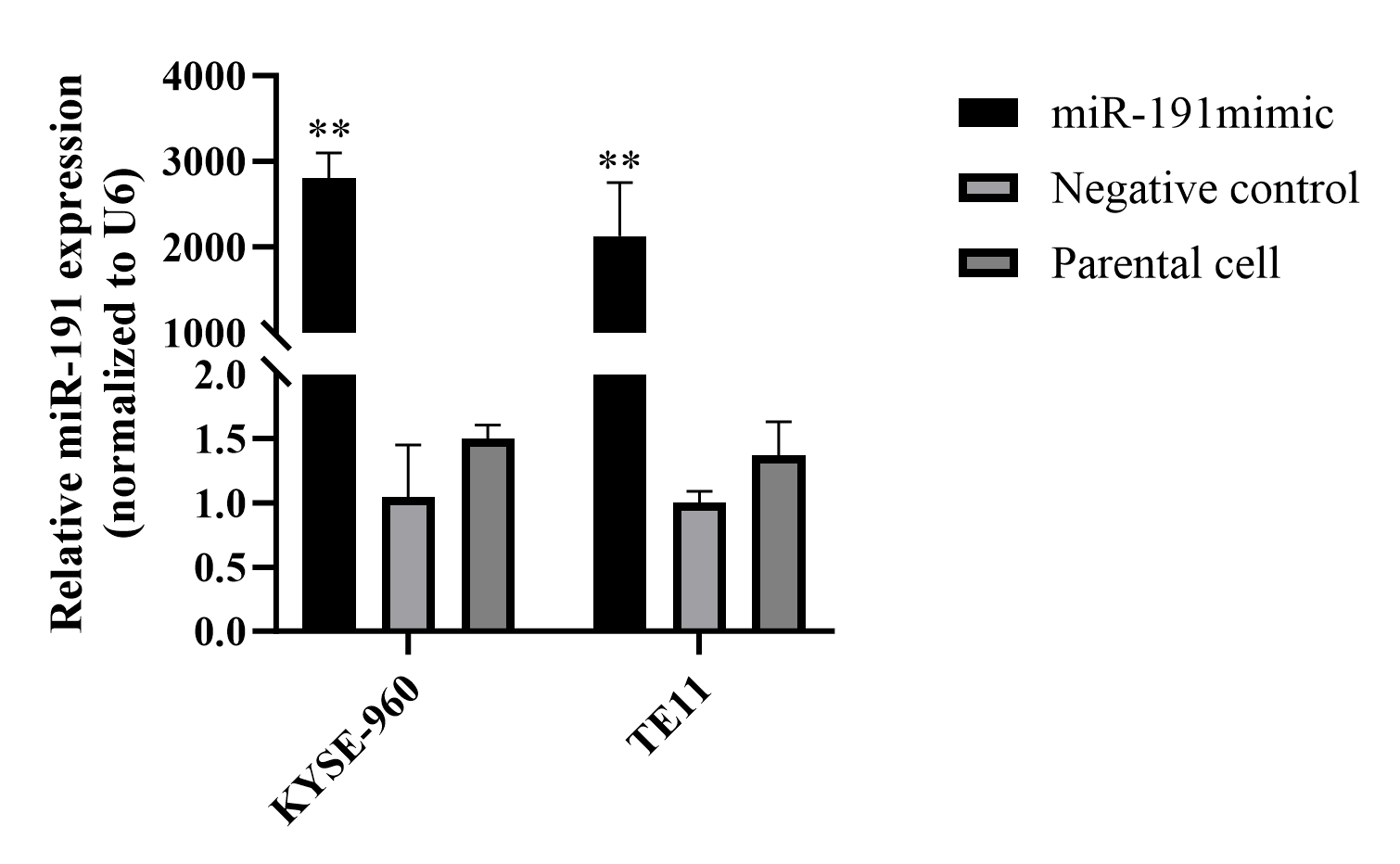
Figure S2.** qRT-PCR of miR-191-5p expression in TE11 and KYSE-960 transfected miR-191-5p or negative control, and parental cells. The cell lines TE11 and KYSE-960 were seeded into 6-well plates (2.5×10^5^ cells per well), and after 24 h, cells were transfected with miR-191 mimic or negative control mimic using Lipofectamine^TM^ RNAi-MAX.


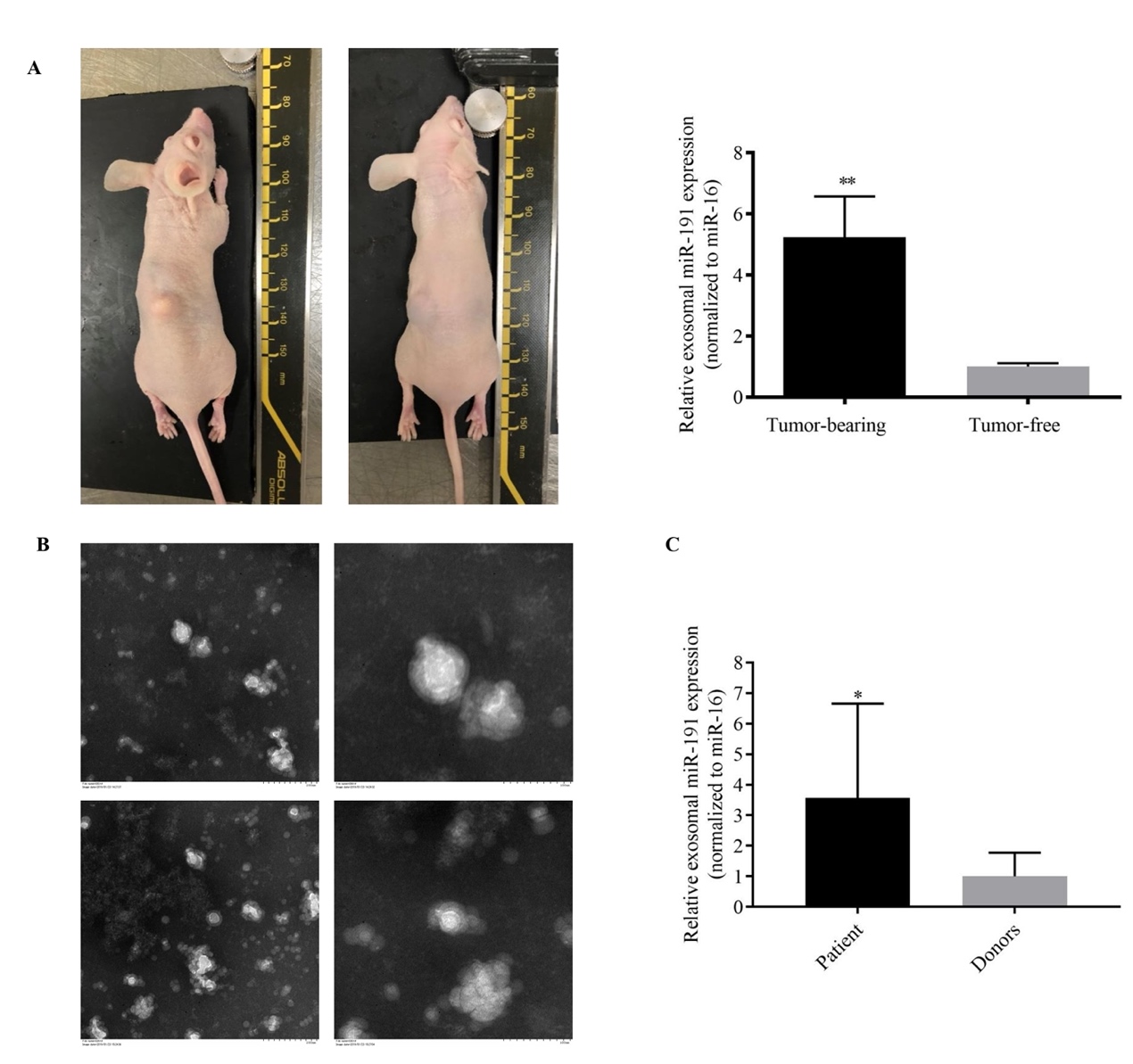


**Figure S3.** miR-191 was upregulated in the tumor-derived exosomes *in vivo*. A. The expression of exosomal miR-191-5p was significantly higher in the tumor-bearing group than in the tumor-free group. B. An electron microscope image of exosome isolated from the plasma of an esophageal cancer patient. Note the presence of exosomes ranging in size from 30-100 nm. C. The overexpression of miR-191 in exosomes isolated from the plasma of ESCC patients. The miR-191 expression was analyzed by qRT-PCR and normalized to the miR-16 expression. * p=0.0481 and **P < 0.01.

**
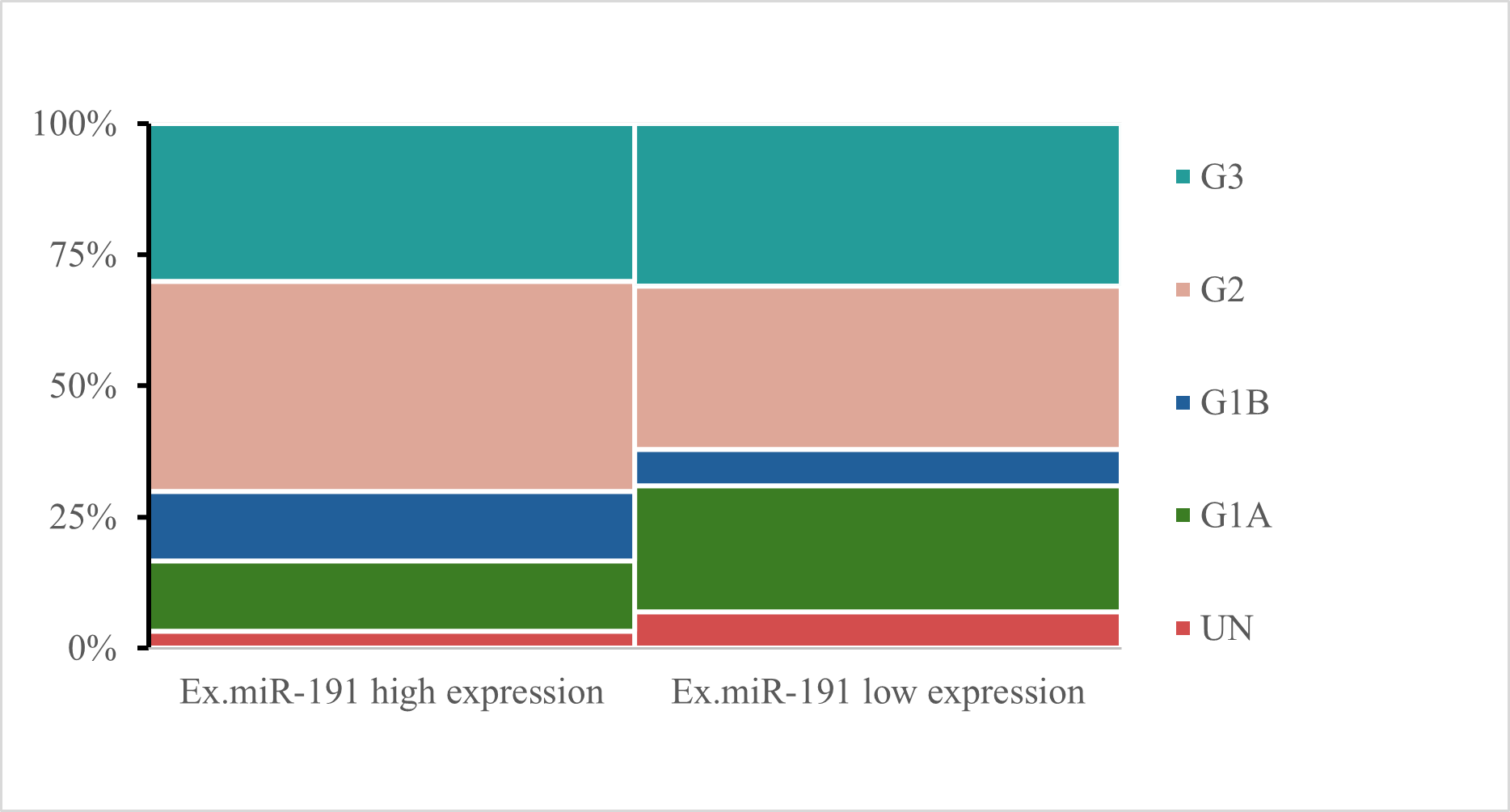
**

**Figure S4.** exosomal miR-191-5p and histological Grade by CRT followed by esophagectomy.

miR-191-5p High（n=29）and miR-191-5p Low(n=30)
