# Supplementary material for: Circulating exosome-derived miR-191-5p is a novel therapeutic biomarker for radiotherapy in esophageal squamous cell carcinoma patients
Source: Esophagus. 2025 Mar 10;22(3):454–66. doi: 10.1007/s10388-025-01116-9 (PMC12167317; doi:10.1007/s10388-025-01116-9)
Supplement: Supplementary file 5 — Supplementary file5 (DOCX 21 KB) [file 10388_2025_1116_MOESM5_ESM.docx]

**Clinical plasma samples**

The plasma samples were collected from 67 patients with newly diagnosed ESCC at Chiba University Hospital (Chiba, Japan) between May 2011 to April 2017 and 6 healthy donors from Chiba University between January 2018 to January 2019: The healthy donors were eligible for this study if they (1) did not have a uncontrolled chronic disease (cardiovascular disease including hypertension, diabetes, chronic respiratory diseases such as chronic obstructive pulmonary disease with impaired oxygenation or ventilation etc.) (2) did not smoke and (3) did not have any cancer. Blood examinations and sampling were performed before treatment. The samples were snap frozen in liquid nitrogen and stored at -80℃. The present study was approved by the Ethics Committee of Graduate School of Medicine, Chiba University. Written informed consent was obtained from all of the patients.

We retrospectively investigated the patients as an initial treatment with or without esophagectomy (with two- or three-field lymph node dissection, if necessary) between 2011 and 2017. The patient medical records and survival status were retrospectively reviewed in August 2018. Patients were deemed eligible for this study if they (1) had been pathologically diagnosed with ESCC, (2) were between 20 and 85 years of age, and (3) did not have any other kinds of cancer. The data of 67 patients were retrospectively examined after excluding the patients who did not meet these criteria. Patients were divided into a high-expression group (n=33) and low-expression group (n=34) according to the median expression of miR-191-5p (cut-off value≥2.5). Two clinical survival outcome endpoints were chosen for the endpoints analysis: disease-specific survival (DFS) and Progression-Free Survival (PFS).

The TNM system (UICC, 8th edition) was used to classify the ESCC stages in these patients. The TRG-PT was classified into 5 categories according to the ratio of viable cancer cells per tumor tissue, as follows: grade 0, no therapeutic effect; grade 1a, viable cancer cells ≥2/3; grade 1b, 1/3 ≤ viable cancer cells <2/3; grade 2, viable cancer cells <1/3; and grade 3, no viable cancer cells(1).

**Extraction of exosomes from the plasma.**

Each plasma sample was centrifuged at 2,000 g for 20 min at room temperature to remove cells and debris. The supernatant containing the partially clarified plasma was transferred to a new tube and then centrifuged at 10,000 g for 20 min at room temperature. The supernatant was then transferred to a new tube, and proteinase K (Proteinase K For Total Exosome Isolation from plasma; Invitrogen, Carlsbad, Calif., United States) was added to remove the endogenous nucleases. Exosomes were isolated using an exosome isolation reagent (Total Exosome precipitation reagent from plasma; Invitrogen) according to the manufacturer's protocol.

**Transmission electron microscope (TEM) observation**

TEM observation was performed using a carbon-coated copper grid (Excel support film, 200 mesh, RL26A; NISSHIN EM Co., Ltd., Tokyo, Japan) with the negative stain method. Negative staining used a 2% phosphotungstic acid solution (pH 7-7.4). All sample grids were subjected to hydrophilic treatment with glow-discharged treatment before absorption using plasma etching device (SEDE-GE; Meiwafosis Co., Ltd., Tokyo, Japan). One dispersed droplet of the sample and two droplets of phosphotungstic acid solution were prepared on a parafilm consisting of 20 μl each, respectively. The stain protocols were the same as the sample absorption protocols, with staining performed twice. After negative staining, the grids were left to dry in a dry box overnight. The samples were then subjected to TEM observation (H-7650; Hitachi High-Technologies Corporation, Tokyo, Japan) at an acceleration voltage of 80.0 kV.

**Western blot analyses**

Cell lysates were obtained using RIPA buffer. Protein was quantified using the BCA protein assay (BIO-RAD, Hercules, CA). In brief, 40 μg of protein was loaded onto a 7.5% to 15% polyacrylamide gel (XV PANTERA GEL; DRC Perfect NT gel System Products, Kyoto, Japan) and then transferred onto a 0.2-μm PVDF membrane (Trans-Blot Turbo^TM^ Transfer Pack; BIO-RAD) and blocked with Tris-buffered saline (TBS)/Tween 20 with 5% non-fat milk or 3% BSA. The membrane was then incubated with the primary antibodies of CD63 (1:1000), CD81 (1:1000), DPAK1 (1:500), ERK (1:500), p-ERK (1:250), JNK (1:500), p-JNK (1:250), p-38 (1:500), p-p38 (1:250), caspase3 (1:500), bcl-2 (1:500, all from Santa Cruz Biotechnology, Santa Cruz, CA, USA) and beta-actin (1:2000, Abcam), GAPDH (1:2000, Abcam) at 4 ℃ overnight and with the HRP-conjugated secondary anti-mouse or rabbit antibody (Sigma, St. Louis, MO, USA) for 30 min at room temperature. Immune complexes were detected using a Chemiluminescence CCD Imaging System (AE-9300 Ez-Capture MG; ATTA CORPORATION, Tokyo, Japan).

**Extraction of exosomes from the cell culture medium**

A total of 5×10^6^ cells were incubated with 10% exosome-free FBS medium for 48 h. A total of 30 ml medium was harvested and centrifuged at 2000 *g* for 30 min to remove cells and debris. Total Exosome Isolation (from cell culture media) reagent (15 ml; Invitrogen, Carlsbad, Calif., United States) was then added to the samples, which were incubated at 4 ℃ overnight. After incubation, the samples were centrifuged at 10,000 *g* for 1 h at 4 ℃. Exosomes were then resuspended in phosphate-buffered saline (PBS) for downstream analyses.

1. Kadota T, Hatogai K, Yano T, Fujita T, Kojima T, Daiko H *et al*: **Pathological tumor regression grade of metastatic tumors in lymph node predicts prognosis in esophageal cancer patients**. *Cancer Sci* 2018, **109**(6):2046-2055.
